# Adherence to anti diabetic medication among patients with diabetes in eastern Uganda; a cross sectional study

**DOI:** 10.1186/s12913-015-0820-5

**Published:** 2015-04-19

**Authors:** James Bagonza, Elizeus Rutebemberwa, William Bazeyo

**Affiliations:** Department of Health Policy, Planning and Management, School of Public Health, Makerere University College of Health Sciences, Kampala, Uganda; Department of Disease Control and Environmental Health, School of Public Health, Makerere University College of Health Sciences, Kampala, Uganda

**Keywords:** Diabetes mellitus, Adherence, Medication, Patients

## Abstract

**Background:**

Lack of adherence to anti diabetic medication causes suboptimal blood sugar control among patients with diabetes and can lead to treatment failures, accelerated development of complications and increased mortality. This study assessed factors associated with adherence to anti diabetic medication in rural eastern Uganda.

**Methods:**

A cross sectional study was conducted among 521 patients with diabetes in Iganga and Bugiri hospitals between October 2012 and January 2013. Respondents were patients who were18 years and above and had been on diabetic treatment for not less than a month. Pretested questionnaires were used. Variables that were collected included socio-demographic characteristics, possible barriers to adherence, and self management efforts. Adherence was assessed using self reports. Descriptive and inferential statistics were done to determine adherence to anti diabetic medication and the associated factors.

**Results:**

The level of adherence to anti diabetic medication was 83.3% and factors that were independently associated with adherence were; having been on anti diabetic drugs for at least three years (OR = 1.89, 95% CI = 1.11 - 3.22), availability of diabetic drugs (OR = 2.59, 95% CI = 1.54 - 3.70), and having ever had diabetic health education (OR = 4.24, 95% CI =1.15 - 15.60).

**Conclusion:**

About four in five patients adhere to anti-diabetic treatment. Strategies aimed at improving anti diabetic drug availability and providing health education could improve adherence.

## Background

Diabetes Mellitus (DM) is a growing public health problem worldwide with an estimated 177 million people affected in 2003, 221 million by 2010 and is expected to rise to 300 million in 2025 with biggest increases in Asia and Africa [1-4]. By 2025, it is estimated that more than 75% of people with diabetes will reside in low income countries [4]. Uganda is experiencing a marked upsurge of diabetes [5,6]. In a random sample from Kampala the capital city and its neighboring district Mukono, the prevalence was estimated to be up to 8.1% [7].

A key dimension of healthcare quality is adherence to prescribed medications. According to the World Health Organization (WHO), adherence is the extent to which a persons behavior; taking medication, following a prescribed diet, and/or executing lifestyle changes corresponds with agreed recommendations from the health care provider [8]. However, non-adherence to medication is particularly common among patients with diabetes [9] and inadequate adherence compromises safety and treatment effectiveness, leading to increased mortality and morbidity. This ultimately translates into significant direct and indirect costs to the healthcare system [10-12]. It is also argued that, because the magnitude of non-adherence and the scope of its effect are high, more health benefits worldwide would result from improving adherence to existing treatments than by developing new medical treatments [8]. Effective management of diabetes is associated with lower morbidity, mortality, and health care utilization hence reducing the cost and burden to government and the community. A study done in United States indicated that people with diabetes who did not adhere to treatment had significantly worse clinical outcomes compared to patients who adhered to treatment [13]. Despite the emphasis put to patients on the importance of appropriate medication use, many patients poorly adhere to drugs especially for chronic diseases [14] and fall short of achievable health goals as a result. Non-adherence is associated with factors that are patient-centered, therapy-related, or healthcare system related [13,15]. The patient-centered factors can be demographic (age, gender, educational level, and marital status) and psychological (patients beliefs and motivation towards the therapy, negative attitude, patient-prescriber relationship, understanding of health issues, and patients knowledge) [16,17]. The therapy-related factors include route of medication, duration of treatment, complexity of treatment, type of medication and the side effects of the medicines. The factors linked to the healthcare system include availability and accessibility of health care, and the health provider-patient interactions [12].

Previously, numerous studies have explored potential risk factors of non adherence to medicines across a variety of conditions [12,18,19]. Frequently cited risk factors include age, sex, ethnicity, income, education, and co-morbidity though their relationship to adherence has been inconsistent due to variations in study designs and sample populations [9,20,21]. Medication related side effects are also associated with non-adherence [22]. A study done in Uganda in an urban hospital indicated long time interval to a facility visit, patients not understanding the drug regimen and inability to afford the cost of the drugs as associated with non adherence [23]. Use of a diet plan and being told why to control diet are also associated with adherence [24].

In Uganda, there is scarcity of literature on adherence to diabetic treatment in rural areas whose population is generally poorer with less access to health care. The number of patients with diabetes in rural areas is on the increase [25]. Therefore there is a need to identify factors related to medication adherence. Furthermore, most of the studies have been carried out in developed countries [9], leaving a gap in knowledge about the prevalence and factors that may be associated with adherence to diabetic treatment in rural settings. In this study, we assessed factors associated with adherence to anti diabetic medication in eastern Uganda so as to guide interventions for improving drug adherence and optimal glycaemic control among patients with diabetes in rural areas.

## Methods

### Study setting

The study was conducted in two general hospitals – Iganga and Bugiri. These hospitals operate out-patients diabetic clinics once a week and have in-patient facilities for the very ill patients. Patients attend the clinics at scheduled times for continuous monitoring and consultation regarding their illness. Medical care is provided throughout the week and patients receive free medical care including medicines and laboratory tests when available. A more detailed description of the study setting has already been described in a previous paper [26].

### Study respondents and data collection

This was a cross sectional study. Respondents were all patients with diabetes, aged at least 18 years, attending the diabetic clinics who gave informed consent to participate in the study. We excluded those who were very ill and those who were newly diagnosed with diabetes (less than one month) from the study. Our outcomes were adherence to anti diabetic medication and the associated factors.

Out of the 575 registered patients with diabetes, a total of 521 patients who reported between October 2012 and January 2013 in these hospitals were recruited consecutively on diabetic clinic days. Both clinics were allocated 12 weeks for patient selection and data collection was done concurrently.

Trained interviewers used pretested semi-structured questionnaires to collect information on: age, gender, education level, marital status, and occupation. Other variables included perceived benefits of treatment, duration on drugs, type of drugs taken, availability of drugs, having had side effects, time since patient was last seen by health worker, use of alternative medicine, having ever had diabetic education, understanding the drug regimen and having received reminders to take drugs.

### Assessment of adherence to anti diabetic medication

Therapeutic adherence means that the patient observes the medical recommendations, taking the medication, and maintaining a lifestyle as recommended by clinicians [18]. In our study, we focused on medication adherence. Adherence to diabetic treatment was determined through self reports of how patients had been taking medication one week prior to the interview. Patients were specifically asked to recall if they missed any doses of medication on a day by day basis over a period of seven days. The number of times doses were missed was calculated basing on the patients medication regimen which was obtained from their medical forms.

### Operational definition of adherence to anti diabetic medication

We considered patients who took 80% and above of the prescribed doses over the last seven days as adherent to anti diabetic drugs [12,23,27].

### Data management and analysis

Data was entered using double data entry, cleaned to ensure completeness and consistency and checked for normality. Univariate analysis was used to summarize data, describe social demographic characteristics of study respondents and determine level of adherence. We used bivariate analysis to determine associations between adherence and various independent factors using STATA 10.0 statistical software (Stata, College Station, TX, USA). Crude odds ratios with their corresponding 95% confidence intervals were used to describe these associations. Independent variables with p-values less than 0.25 were used for multinomial analysis to determine variables which were independently associated with medication adherence. Independent factors were determined through a logistic regression analysis with stepwise elimination method while keeping known predictors of adherence from literature, in the multivariate model.

### Ethical considerations

Ethical approval to conduct this study was obtained from Makerere University School of Public Higher Degrees Research and Ethics Committee and the Uganda National Council for Science and Technology. Permission was granted by Iganga and Bugiri district health authorities. Written informed consent was obtained from study participants. Data accruing was kept securely locked at all times and was only accessible to researchers.

## Results

### Participants characteristics

A total of 521 diabetic patients were interviewed of whom 259 (49.7%) were female. The mean age was 50.9 years (standard deviation 14.6 years). Nearly three-quarters, 381 (73.1%) were married, and about half, 238 (45.7%) had no formal education. Details of the respondents background characteristics are summarized in Table 1.

**Table 1:** **Socio-demographic characteristics of respondents**

**Characteristics**	**Frequency [n,(%)]**
**Age**	
<35	72 (13.8)
36 - 45	113 (21.7)
46 - 55	153 (29.4)
>55	183 (35.1)
**Sex**	
Female	259 (49.7)
Male	262 (50.3)
**Education**	
None	238 (45.7)
Primary	179 (34.4)
Secondary	73 (14.0)
Tertiary	31 (5.9)
**Marital Status**	
Married	381 (73.1)
Separated	41 (7.9)
Single	30 (5.8)
Widowed	69 (13.2)
**Occupation**	
Casual laborer	16 (3.1)
Farmer	275 (52.8)
Government employment	42 (8.1)
Trader	91 (17.5)
House wife	25 (4.8)
Unemployed	41 (7.9)
Others	31 (5.9)
**Hospital**	
Bugiri	221 (42.4)
Iganga	300 (57.6)

### Adherence to anti diabetic medication

Of the 521 participants, 433 (83.3%) were adherent to anti-diabetic medications based on an adherence index of 80% from self reports [12,23,27]. Respondents socio-demographic characteristics such as age, sex, education level and marital status were not associated with adherence to anti diabetic medication.

### Factors associated with adherence to diabetic medication

At crude analysis, factors associated with adherence to medication were: being on anti diabetic drugs for at least three years (OR = 2.25, 95% CI = 1.38 – 3.66), receiving anti diabetic drugs in injection form (OR = 1.78, 95% CI = 1.07 – 2.96), availability of anti diabetic medication (OR = 2.56, 95% CI = 1.56 – 4.16), not using alternative medicine (OR = 2.26 95% CI = 1.33 – 3.83), having ever had diabetic education (OR = 3.33, 95% CI = 1.06 – 11.1) and having no reminders to take medication (OR = 0.25, 95% CI = 0.10 – 0.56). Details are summarized in Table 2.

**Table 2:** **Factors associated with adherence to anti diabetic medication**

**Variable**	**Adherence**		**OR (95% CI)**	**p-value**
	**Yes [n,(%)]**	**No [n,(%)]**		
**Perceived benefits of DM drugs**				
A lot of benefits	251 (85.9)	41 (14.1)	1.00	
Some benefits	182 (75.2)	42 (24.8)	0.64 (0.40 – 1.02)	0.063
**Duration on DM drugs**				
Less or equal to 3years	198 (77.6)	57 (22.4)	1.00	
More than 3 years	235 (88.7)	30 (11.3)	2.25 (1.38 – 3.66)	<0.001
**Type of DM drugs taken**				
Orals only	241 (80.3)	50 (19.3)	1.00	
Injections only	182 (87.9)	25 (12.1)	1.78 (1.07 – 2.96)	0.024
Both oral and injections	10 (81.8)	3 (18.2)	1.10 (0.23 – 5.24)	0.903
**Do you get all the prescribed drugs**				
Sometimes	133(74.2)	43 (25.8)	1.00	
Always	297 (87.9.)	41 (12.1)	2.56 (1.56 – 4.16)	0.001
**Ever experienced side effects**				
Yes	56 (76.7)	17 (23.3)	1.00	
No	365 (84.9)	65 (15.1)	1.70 (0.93 – 3.12)	0.080
**Time when last seen by the health worker**				
Less or equal to one month	211 (82.4)	45 (17.6)	1.00	
More than one month	214 (84.6)	39 (15.6)	1.17 (0.73 – 1.87)	0.430
**Use of alternative medicine**				
Yes	53 (69.7)	23 (30.3)	1.00	
No	367 (85.9)	60 (14.1)	2.65 (1.50 – 4.68)	<0.001
**Ever had diabetic education**				
No	8 (61.5)	5 (38.5)	1.00	
Yes	421 (84.2)	79 (15.8)	3.33 (1.06 – 11.10)	0.029
**Understanding the drug regimen**				
Yes	415 (83.5)	82 (16.5)	1.00	
No	16 (73.7)	7 (26.3)	0.55 (0.19 – 1.58)	0.262
**Reminders to take drugs**				
Radio/TV	87 (86.1)	14 (13.9)	1.00	
Watch/clock	126 (84.0)	24 (26.0)	0.84 (0.41- 1.72)	0.643
Phones	73 (84.9)	13 (15.1)	0.90 (0.39 - 2.04)	0.808
Shadows	8 (88.9)	1 (11.1)	1.28 (0.14 – 11.2)	0.818
Others	108 987.1)	16 (12.9)	1.08 (0.50 – 2.35)	0.833
None	30 (61.2)	19 (38.8)	0.25 (0.10 – 0.56)	0.006

### Independent factors associated with adherence to diabetic medication

Factors independently associated with adherence to anti diabetic medication after adjusted analyses were: having taken medication for more than three years (OR = 1.89, 95% CI = 1.11 – 3.22), availability of anti-diabetic drugs (OR = 2.59, 95% CI = 1.54 – 3.70), and ever had diabetic education (OR = 4.24, 95% CI = 1.15 – 15.60). Details are summarized in Table 3.

**Table 3:** **Logistic regression analysis of predictors of medication adherence**

**Variable**	**Crude OR (95% CI)**	**Adjusted OR (95% CI)**	**p value**
Duration on DM more than 3 years	2.25 (1.38 – 3.66)	1.89 (1.11 – 3.22)	0.018
Having got anti diabetic drugs always	2.56 (1.06 – 4.17)	2.59 (1.54 – 3.70)	<0.001
Not using alternative medicine	2.26 (1.33 – 3.83)	1.73 (0.92 – 3.28)	0.090
Having ever had diabetic education	3.33 (1.06 – 11.10)	4.24 (1.15 – 15.60)	0.030
Using injectable anti diabetic drugs	1.78 (1.07 – 2.96)	1.27 (0.73 – 2.21)	0.399
Never experienced anti diabetic drug side effects	1.70 (0.93 – 3.12)	1.38 (0.69 – 2.75)	0.367

## Discussion

In this study, adherence to anti diabetic medication was 83.3% and this was associated with duration on anti-diabetic treatment, drug availability and having ever had diabetic education.

About four in every five respondents adhered well to their diabetic medication based on self reports at an adherence index of 80% [12,23,27]. Since adherence information was based on patients recall, the actual adherence levels may be lower than what was found in our study. This is because self-reports usually overestimate patients adherence levels [23]. Furthermore, patients may get problems recalling how they took medication but this was minimized by asking them to recall over a short period of only one week. Thirdly, potential response bias was minimized by asking non critical and non threatening questions when assessing for medication adherence. Similar rates of adherence have been reported previously [9,11,12,17]. However, low rates of adherence to diabetic medication have also been reported in India, Malaysia, Saudi Arabia and Korea [18,27-29]. The discrepancy in adherence levels may be attributed to differences in metrics to assess adherence, and/or differences in health care settings and socio economic status. With reference to our study, patients receive free anti diabetic drugs while in countries like India, Malaysia and Korea, patients pay for drugs and clinic consultations. These associated financial costs may deter or delay patients from re-filling prescribed medication and this negatively impacts on their adherence. Secondly the high costs of the prescribed oral hypoglycemic medications especially the relatively newer agents or scarcity of prescribed brands of medications further hinders optimal adherence [11,30]. Financial costs associated with diabetic care whether direct or indirect significantly reduce access to diabetic therapy and thus influence patients adherence in developing countries [18,31,32].

In our multivariate model, respondents who had been on anti diabetic medication for at least three years significantly had high levels of adherence (AOR 1.11 – 3.22). Similar results were observed from a study among diabetic patients in France, which showed that patients with poor adherence had been on diabetic treatment for less than five years [33]. Patients with longer durations of diabetic medication are likely to have had more interactions with their health care providers, could have understood their regimen better and would be self motivated to take their medication. It is possible that patients who have been on treatment for a short duration may be less aware of their disease and are thus more likely to be non adherent. Alternatively, it could be that the adherence to treatment may be responsible for patients lasting longer than three years. A prospective study assessing adherence of patients with diabetes would be useful to determine to what extent adherence contributes to their survival.

Unavailability of anti diabetic medication was associated with non adherence (AOR 1.54 – 3.70). Nearly a third, 33.8% of the respondents reported having missed getting at least one of the drugs in their regimen from the hospitals in the previous two months. Other studies have reported anti diabetic drugs stock-outs as a major constraint in managing diabetes [23,34-37]. The situation is more critical for those needing insulin. Insulin is much less available and less affordable compared to the oral hypoglycemic medicines. Access to insulin remains poor in many regions of the world due to high prices, exposing patients to risk of serious complications and disease, such as blindness, amputation and death. Insulin prices in private pharmacies vary considerably between regions and between countries within the same region [34]. These findings imply that many patients would go without drugs till they are able to purchase their medications or the next scheduled visit with their health care provider. Failure to afford medications is the most common reason for poor adherence [23]. In our study the hospitals provide free medications for only one month. Sometimes appointments are longer than a month and thus patients are forced to refill their prescriptions from private drug shops and pharmacies. Unfortunately in rural areas, few patients can afford such price. There are also fewer facilities offering anti-diabetic medication in rural areas compared to urban areas. Some patients resort to taking traditional medicines [26,38,39]. Consequently this negatively impacts on their adherence. Irregular supply for anti diabetic drugs has been linked to underfunding and supply chain problems in most health care systems [35,40].

In this study diabetic health education was significantly associated with adherence to medication (AOR 1.15 – 15.60). This is in agreement with other studies that have reported similar associations in Europe, United States and Africa [31,41,42]. Sensitization about the disease, the diagnosis and management of diabetes is effective in improving recruitment of patients into treatment programs as well as improving drug adherence [43]. Diabetes self-management education has the potential to improve diabetes care [43]. Meeting with a diabetes educator for 30 minutes every three months before an office visit may help to achieve many goals [44]. The use of educational materials, regular interviews, instructions from nurses and physicians about methods of incorporating drug administration into patients daily lives, a real partnership between physician and patient, and patient self management of diabetic treatment, have all been found to improve adherence [45]. It is critical therefore that efforts be put in equipping diabetic clinics with adequate numbers of well trained health workers current with the new diabetic management guidelines and willing to spend reasonable time with patients [46,47].

### Methodological considerations

The use of self reports has limitations as an estimate of patients adherence levels. Self reports might be subjective and may overestimate patients adherence status when compared to other methods such as pill counts, prescription claims or biological assays [48,49]. However, the assessments for adherence using self reports may make patients feel comfortable in telling the truth and may probably facilitate the identification of poor adherence [50,51], and thus, it is a reliable and cost-effective method. This method of assessing adherence has its benefits, since it helps health care providers to identify patients who are more likely to have poor adherence, and determine aspects of diabetes that they can focus on to improve patients management [52].

## Conclusions

Our findings showed that only four out of every five patients adhered to anti diabetic medication in a rural setting. Adherence was associated with duration on anti diabetic medication, availability of drugs, and having received diabetic health education. Patients who had been in diabetic treatment for more than three years were more likely to adhere to treatment. Therefore strategies to improve anti diabetic drug availability, health education on diabetic care and self management may help in improving adherence levels among patients with diabetes.
